# Parallel Computing Sparse Wavelet Feature Extraction for P300 Speller BCI

**DOI:** 10.1155/2018/4089021

**Published:** 2018-10-02

**Authors:** Zhihua Huang, Minghong Li, Yuanye Ma

**Affiliations:** ^1^College of Mathematics and Computer Science, Fuzhou University, Fuzhou, China; ^2^Department of Physiology, Yunnan University of Traditional Chinese Medicine, Kunming, China; ^3^Kunming Institute of Zoology, CAS, Kunming, China

## Abstract

This work is intended to increase the classification accuracy of single EEG epoch, reduce the number of repeated stimuli, and improve the information transfer rate (ITR) of P300 Speller. Target EEG epochs and nontarget EEG ones are both mapped to a space by Wavelet. In this space, Fisher Criterion is used to measure the difference between target and nontarget ones. Only a few Daubechies wavelet bases corresponding to big differences are selected to construct a matrix, by which EEG epochs are transformed to feature vectors. To ensure the online experiments, the computation tasks are distributed to several computers that are managed and integrated by Storm so that they could be parallelly carried out. The proposed feature extraction was compared with the typical methods by testing its performance of classifying single EEG epoch and detecting characters. Our method achieved higher accuracies of classification and detection. The ITRs also reflected the superiority of our method. The parallel computing scheme of our method was deployed on a small scale Storm cluster containing three desktop computers. The average feedback time for one round of EEG epochs was 1.57 ms. The proposed method can improve the performance of P300 Speller BCI. Its parallel computing scheme is able to support fast feedback required by online experiments. The number of repeated stimuli can be significantly reduced by our method. The parallel computing scheme not only supports our wavelet feature extraction but also provides a framework for other algorithms developed for P300 Speller.

## 1. Introduction

The advances in the field of brain-computer interfaces (BCIs) are encouraging researchers to explore its various possibilities of benefiting the studies of neuroscience, artificial intelligence, biomedical engineering, and so on. A BCI is a system that measures the activity of central nervous system (CNS) and converts it to artificial output which can change the interactions between the brain and its environment [1]. Although the activity of CNS can be measured by all kinds of brain signals, most BCIs rely on electrical measures because they can be acquired easily [1, 2]. Electroencephalogram (EEG) is the electrical measure of the activity of CNS from electrodes placed on the scalp. BCIs based on EEG are noninvasive. Because of this, the prospective applications of this kind of BCI on persons, including special patients and everyman, impress the researchers in the area.

The EEG-based BCIs could be fulfilled in diverse ways, which mainly include P300 Speller, sensorimotor rhythm (SMR), steady-state visual evoked potential (SSVEP), and slow cortical potential (SCP) [1–4]. They rely on different neuroscience principles and have different features. P300 Speller BCIs were first proposed in [3] and constructed on the basis of P300 event-related potentials (ERP) that are evoked by the target stimulus in an oddball paradigm and mean that the subjects are paying attention to the target [1–4]. An advantage of P300 Speller BCIs is that only a few trainings enable subjects to use them to spell words to a computer and achieve a stable performance [1, 2]. Many researchers are attracted by the potential of P300 Speller BCIs to seek the possible improvements and their applications [1, 5–10]. In the research [5], an asynchronous BCI, which is able to automatically detect the intention of subjects starting to spell words, was designed by combining P300 and SSVEP. It was reported that a system using dynamic P300 Speller matrix can facilitate the access of severely motor-impaired persons to the World Wide Web and multimedia content [6]. According to [7], some researchers explored the approach to improving the P300 Speller performance by using a green family faces paradigm. Krumpe and colleagues considered the situation where a stimulus-locked classification cannot be used. They evaluated the feasibility of detecting a P300 in a reactive EEG-based BCI through an asynchronous classification in their study [8]. Jin et al. observed the decrease of subjects' attention during the presentation of stimuli in P300 Speller BCI and proposed to use honey-comb-shaped figures with 1–3 red points as stimuli to catch subjects' attention [9]. According to [10], Mao et al. reviewed the progress of the application of EEG-based BCI to interaction with robots and enumerated many promising examples of applying P300-based BCI.

To implement P300-based BCI, the key is the detection of P300 ERP. As for ERP estimation, averaging many EEG epochs is the most common practice. Although this method can serve the implementation of P300-based BCI, it faces very big challenges because the stimulus is needed to be repeated many times. It is hard to improve the response time and information transfer rate (ITR) of P300-based BCI if averaging EEG epochs underlies the detection of P300 ERP. As a result, many approaches based on machine learning have been developed for P300 detection in this kind of BCIs. They usually consist of a few important steps such as extracting the features from EEG and training an appropriate classifier. Since EEG or ERP are the signals acquired from the scalp, wavelet analysis, one of excellent signal processing tools [11], is very suitable to be used to handle the problem of extracting the features from EEG.

According to [11], unlike the Fourier transform, whose basis functions, sine or cosine, are all in a frequency and extend infinitely in time, wavelet analysis is based on completely different basis functions that are localized in time and frequency. The ability of wavelet analysis to highlight specific time and frequency components of signals makes itself very useful for processing EEG or ERP [12, 13]. Some examples of applying wavelet analysis in the field include EEG spike detection, ERP component separation, denoising EEG or ERP, etc [12, 13]. Demiralp et al. discovered that the delta response dominates the P300 component in their research and then used the wavelet coefficients of EEG epochs in the frequency range of 0.5–4 Hz during the time period of 310–430 ms after stimulus onset to detect P300 and found that the cognitive state could influence the presence of P300 [14, 15]. Perseh and Sharafat developed a scheme that extracts the features from EEG epochs for P300 Speller BCI by applying wavelet transform [16]. In [17], Robinson and colleagues showed that the wavelet-common spatial pattern algorithm could effectively extract informative features from EEG data for classifying the two different speeds of the right-hand movement. Aniyan et al. designed an algorithm to separate ERP components in single-trial EEG data by making use of the asymmetry of wavelet [18]. Huang and Zheng [19] presented a method to process P300 ERP on the basis of the combination of autoregression model and wavelet representation.

Wavelet methodology has had a significant impact in the area of time series [11] and has been extensively used to deal with all kinds of problems on EEG and ERP [12–19]. In this paper, we use wavelet analysis to address the issue of EEG feature extraction in P300 Speller BCI. According to [3, 20], in P300 Speller BCI, a target EEG epoch is among the consecutive EEG epochs that are overlapped on one another so that nontarget EEG epochs could also contain P300 waveform just in a bit different time range, compared to target EEG epochs. Hence, it is still difficult to highlight the difference between target EEG epochs and nontarget EEG epochs simply using wavelet in P300 Speller BCI. Here, we proposed an algorithm to extract informative features from EEG epochs for P300 Speller BCI by combining wavelet analysis with Fisher Criterion [21]. The idea underlying this algorithm is that the ERP evoked in P300 Speller is a kind of sparse signal in the wavelet domain. So, we call it sparse wavelet feature extraction. Considering the speed requirement of the online experiments, we also designed and implemented the parallel computing scheme of the algorithm based on our previous work [22] and the real-time distributed computation platform of storm [23, 24]. We tested our algorithm in the experiment, and the results demonstrated its effectiveness. This work was partly presented in the conferences [25, 26].

## 2. Materials and Methods

The P300 Speller paradigm [3, 20] intuitively tells us that the difference between target EEG epochs and nontarget EEG epochs is not obvious. Averaging enough EEG epochs could highlight the difference. However, it would be accomplished only at the cost of time and efficiency. Our intention is to seek a way in which the features substantially reflecting the difference could be extracted from single-trial EEG epochs. The key of our method is to find the sparse wavelet bases for P300 Speller BCI. The algorithm is implemented on Matlab.

### 2.1. Wavelet Transform

Wavelet transform can explore the details of a signal in different scales at any time position. Formally, the wavelet transform of a time signal *f*(*t*) is defined as following [11]:

(1) $$\begin{array}{c} C\left(a,b\right)={\int^{}}_{-\infty }^{+\infty }f\left(t\right)\psi_{a,b}^{*}\left(t\right)\text{dt,} \end{array}$$

where $\psi_{a,b}\left(t\right)=1/\sqrt{a}\psi \left(\left(t-b\right)/a\right)$, *a* > 0, *b* ∈ *R*, and *∗* means complex conjugation.

Equation (1) shows that wavelet transform maps a function of time to another function of *a* and *b*, which, respectively, represent scale and time location. So, local frequency information of signals can be reflected clearly by wavelet transform. This is very import to P300 Speller BCI that need to find the time and frequency range of EEG epochs during which the differences between target and nontarget exist. The direct numerical implementation of Equation (1) usually is called continuous wavelet transform (CWT) [11]. However, CWT involves too many closely spaced scales and time points that are highly correlated. The information provided by CWT is unnecessarily redundant and CWT is not very efficient. A computationally simpler implementation is discrete wavelet transform (DWT) that is constructed on a set of orthogonal wavelet bases [11], such as Daubechies Wavelet [12]. DWT algorithm is based on a simple recursive filter scheme. The result of DWT algorithm is not redundant but sufficient for reconstruction of the time function.

In P300 Speller BCI, the *f*(*t*) would be digitally sampled, and in the time window following the stimulus onset, be extracted into an EEG epoch that can be denoted as a vector *e*. DWT transforms *e* to another vector in same dimension. It can be formally written as follows:

(2) $$\begin{array}{c} b=We, \end{array}$$

where *W* is an orthogonal matrix representing DWT, *b* as a vector in same dimension as *e*, is the result of DWT. In *P*300 Speller BCI, the difference between target and nontarget EEG epochs is that *P*300 component appears at different positions of the EEG epochs, so only a few elements reflecting the difference benefit the recognition of target EEG epochs. Selecting them from all elements is helpful.

### 2.2. Fisher Criterion

Clearly, it is not efficient to use all elements of *b* in recognizing target EEG epochs. The next problem we face is to find a way in which only the best elements are selected to be passed to classifiers.

Aniyan et al. [18] reported a wavelet-based method carrying out the detection and isolation of special ERP component. The method is a fully automated algorithm that selects the best scale analysis from CWT for separating ERP components from single-trial EEG epochs. Although the problem handled by [18] is different, it at least demonstrates the feasibility of developing an algorithm for the issue that we need to solve.

According to [21], Fisher Criterion is a discriminant criterion function that is defined by the ratio of the between-class scatter to the within-class scatter. By maximizing the criterion function, one can get a projection axis. After the samples are projected on the axis, the between-class scatter is maximized and the within-class scatter is minimized.

In P300 Speller BCI, the aim is discriminating target EEG epochs and nontarget EEG epochs. We denote an EEG epoch on a channel by a vector *e*. Further, *ae*^+^ represents a target EEG epoch and *ae*^−^ means a nontarget epoch. DWT, respectively, transforms *e*^+^ and *e*^−^ to *b*^+^ and *b*^−^. Some elements of *b* are useful for discriminating *b*^+^ and *b*^−^ but others not. We need to use Fisher Criterion to optimize DWT for P300 Speller BCI. In the optimized DWT, only the elements that clearly benefit the discrimination of the two classes remain in the results of transformation.

### 2.3. EEG Feature Extraction Algorithm

In Equation (2), the matrix represents DWT. In fact, our optimization means removing some rows from *W* in Equation (2). In accordance with Fisher Criterion, we need to maximize a function described by Equation (3) to seek a solution.

(3) $$\begin{array}{c} J\left(\omega \right)=\frac{{\left(\omega^{T}\bar{b^{+}}-\omega^{T}\bar{b^{-}}\right)}^{2}}{\omega^{T}\Sigma^{+}\omega +\omega^{T}\Sigma^{-}\omega }, \end{array}$$

where $\bar{b^{+}}$ and $\bar{b^{-}}$, respectively, represent the means of *b*^+^ and *b*^−^ and Σ^+^ and Σ^−^ are the covariance matrix of *b*^+^ and *b*^−^.

According to [21], the direction of the expected unit vector can be obtained by the following equation:

(4) $$\begin{array}{c} \omega ={\left(\Sigma^{+}+\Sigma^{-}\right)}^{-1}\left(\bar{b^{+}}-\bar{b^{-}}\right). \end{array}$$

In the vector *ω*, the absolute values of the elements mean their importance to discriminating *b*^+^ and *b*^−^. Each element of *ω* corresponds to a row of *W*. This shows that we can sort the elements of *ω* by their absolute values, remove some rows of *W* corresponding to the elements of small absolute values, and then get a new transform matrix *M*. In fact, *M* is a set of optimal wavelet bases for the channel of the subject. On the basis of the above analysis, we propose our EEG feature extraction algorithm for *P*300 Speller BCI. The algorithm includes two stages. In short, the first stage is to get *M* and the second stage is to use *M*.

The goal of the first stage is to determine an *M* for each channel. After training a subject, we can collect some target EEG epochs and nontarget EEG epochs. By them, a data set comprising many *e*^+^ and *e*^−^ can be built for every valuable channel. According to Equation (2), the data set can be transformed to another data set containing many *b*^+^ and *b*^−^. From this new data set, *ω* can be obtained by Equation (4). In light of the absolute value of *ω* elements, the rows of *W* that are important to discriminating the target EEG epochs and nontarget EEG epochs are selected to construct *M*. The first stage of EEG feature extraction algorithm is depicted as Algorithm 1. In this, *N* means that we need to transform EEG epochs on the channel to *N*-dimension feature vectors, Matrix *W* represents the DWT for the data set comprising many *e*^+^ and *e*^−^, and Matrix *M* would be used to compute the feature vectors of EEG epochs on same channel.

In the second stage, every valuable channel of EEG epochs can be transformed to a low dimension vector by the following equation:

(5) $$\begin{array}{c} r=Me. \end{array}$$

All *r* of an EEG epoch can be concatenated into a feature vector, which is in much lower dimension than the EEG epoch but has the most information in the EEG epoch that is helpful to the discrimination of target and nontarget. The feature vector is the result of EEG feature extraction algorithm.

### 2.4. Parallel Computing Scheme

In a P300 Speller BCI, the classifiers are always trained offline when the subjects are in rest. Therefore, the first stage is also carried out offline if EEG feature extraction algorithm is applied in a P300 Speller BCI. Its speed is not a critical factor. As for the second stage, it is performed online when a P300 Speller BCI using the algorithm is running. Every EEG epoch is needed to be computed when it is gotten, and the computation is expected to be completed as soon as possible. So, the speed is very important for the second stage of EEG feature extraction algorithm.

In fact, we have met the speed trouble. Originally, we implemented the algorithm in BCI2000 and tried online P300 Speller experiments of the algorithm. BCI2000 often stopped the experiments since the computation could not keep up with the steps of the experiments.

Although the speed problem can be solved by replacing the desktop computer with a high-performance hardware system, it is not a good solution in view of the cost and convenience. A preliminary parallel computing framework was proposed to increase the computation speed of P300 Speller in our previous work [22]. Here, we also seek to distribute the computing tasks of the algorithm to several desktop computers that are cooperating with one another. A very good mechanism for this problem is Storm, an open source real-time distributed stream data processing system [23, 24]. Its performances of low latency and fault-tolerance have been verified by many famous applications. We designed a parallel computing scheme of EEG feature extraction algorithm on the basis of Storm.

**Figure 1:**
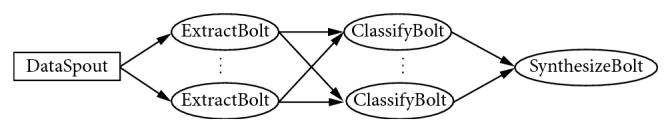
Parallel computing scheme.

The parallel computing scheme of the algorithm based on Storm is shown in Figure 1. The scheme includes four kinds of computing units: DataSpout, ExtractBolt, ClassifyBolt, and SynthesizeBolt. They are built in accordance with the standard of Spout or Bolt in Storm [23, 24]. There is only one DataSpout in the scheme. DataSpout is connected to the EEG acquirement system and receives the signal segments from it. DataSpout assembles an EEG epoch and sends it to an ExtractBolt when the segments that have come are enough for an EEG epoch. The task of ExtractBolt is to conduct the computation described in Equation (5) for each channel of an EEG epoch. There are many ExtractBolts in the scheme. The number of them depends on how many computers and what kind of computer is included in this scheme. All ExtractBolts simultaneously operate but, respectively, compute different EEG epochs. An ExtractBolt outputs a feature vector to a ClassifyBolt when it completes the computation of an EEG epoch. Every ClassifyBolt is a computing unit of classifying a feature vector. Although the classification is not the focus in this paper, the scheme should contain the classification from the perspective of the computation integrity. In this scheme, all ClassifyBolts implement the same classifier. Similar to the ExtractBolts, many ClassifyBolts run at the same time but handle different feature vectors. Every ClassifyBolt transmits the score of classifying the feature vector to SynthesizeBolt. There is only one SynthesizeBolt in the scheme. SynthesizeBolt simply picks the row and column corresponding to the biggest the scores and feedbacks them to other parts of the BCI system.

Usually, one round of stimuli is not sufficient for a satisfactory detection of characters. The scores of a few rounds are necessarily accumulated to enhance the reliability. SynthesizeBolt does not give out the feedbacks until the gap between the biggest score and the second biggest one exceeds a threshold. Since the scores of a few rounds are summed, the computing units in ExtractBolt and ClassifyBolt are required to be linear. Equation (5) shows that the computing units in ExtractBolt are linear. As for ClassifyBolt, nonlinear classifiers are not acceptable. On the contrary, this scheme does not have a preference for any linear classifier. The scheme is flexible at this point.

## 3. Results and Discussion

### 3.1. Preliminary Work

#### 3.1.1. Experiment Design

Our main aim is verifying whether EEG feature extraction algorithm can improve the performance of P300 Speller BCI by reducing the number of repeated stimuli. We designed the experiment procedure according to the P300 speller paradigm [1, 3, 4, 20]. The subjects were instructed to quietly sit in front of a screen and gaze at the screen. A 6 × 6 matrix of characters was presented on this screen. The six rows and six columns of the character matrix were randomly highlighted. The duration of highlighting was 120 ms, and the interval between the two consecutive highlighting was 80 ms. Each of the six rows and six columns was highlighted once in a round. This kind of flash was conducted fifteen rounds, called a sequence, for a wanted character. The subjects were asked to silently count when the wanted characters were being highlighted.

The experiments involved three phases. The first phase aimed to provide the subjects with practice opportunities so that they could adapt themselves to operating P300 speller. The intention of the second phase was to construct a training set for each subject. The goal of the third phase was to build a test set for each subject.

#### 3.1.2. Subject and Instrument

Nine subjects (three males and six females) took part in the experiments. Eight of them were university students (the average age was 24 years old), and one was a university staff (43 years old). All subjects were right-handed, and their eyesight varied in the degree of myopia. All subjects had sufficient rest between the experiments. For convenience, the nine subjects were denoted by S1 to S9.

The instrument for EEG signal acquisition was the 64-channel Neuroscan system, including the EEG cap, the amplifier, and the signal acquisition software. The sampling rate was set to 1000 Hz. BCI2000 [20] was used to present the character matrix and the stimuli, and it was integrated with the Neuroscan signal acquisition software to process the signals and save them to the data files.

#### 3.1.3. Data Set

In the second phase, the following steps were taken to construct the training sets. All characters in the character matrix were randomly divided into four groups. Every subject was arranged to input the characters group by group by the means of P300 speller. The course during which a subject input one group of characters is defined as a run. Between the two consecutive runs, the subjects all had a chance to sufficiently rest.

EEG signals were acquired when the subjects were working. First, the signals were filtered by such preliminary processes such as common average reference (CAR). Next, the signals except those from the electrodes of FZ, CZ, PZ, PO7, PO8, and OZ were removed. The reason for doing so is that the signals from these electrodes evidently have the main effect on P300 speller [1]. Every signal epoch of 800 ms following a stimulus onset was cut out. A stimulus onset corresponds to six 800 ms signal epochs from the six electrodes. The epochs corresponding to the wanted characters were labeled as positive examples and others as negative examples. All positive examples and negative ones belonging to one subject were added into the training set of the subject.

Before the third phase, every signal epoch was downsampled to a 15-dimension vector, then the six vectors corresponding to one stimulus onset were concatenated to be a feature vector, and a linear discriminant function was trained on the set of feature vectors for each subject. In the third phase, the subjects were instructed to formally use P300 Speller. BCI2000 was configured to use the trained linear discriminant functions to recognize the targets. As mentioned before, the subjects input about 10 characters in a run and had a rest after a run. Meanwhile, the EEG signals were stored into the data files for the subsequent analyses.

The most experiments in the third phase achieved the run accuracy of 100% for the character recognition. Although the accuracies of the remaining runs were close to 100%, only the runs with the accuracy of 100% were selected into the test sets. This ensured that the test sets were composed of the best data, and the adverse influence of dirty data could be excluded as much as possible.

### 3.2. Procedure and Performance of Classifying

The proposed method involves both Wavelet Transform and Fisher Criterion, so we call it WF in a brief form. The question on whether or not WF could improve the performance of classifying EEG epochs in P300 Speller BCI is needed to be tested. Since WF does not focus on classifier but feature extraction, WF, downsampling (DS), and xDAWN (xD) [27] were, respectively, combined with the stepwise linear discriminant analysis (SWLDA), the default classifier in BCI2000, to classify the EEG epochs in the test data sets. According to [28], DS and xD are the typical feature extractions used to classify ERP in BCI. We evaluated WF by comparing the three kinds of classification results.

For WF, all kinds of mother wavelets are available. According to [12], the wave shapes of mother wavelets play an important role in this kind of tasks. On basis of this opinion, we got the difference wave shape by subtracting the averaged EEG epoch corresponding to target stimuli from the averaged EEG epoch corresponding to nontarget stimuli and compared it with a variety of mother wavelets. Finally, we chose the Daubechies 4 (DB4) in the study to construct a matrix for each channel of one subject. As for the dimension of feature vector, WF and DS both transformed a channel of one epoch to a 15-dimension vector. The reason for 15-dimension is that, by default, BCI2000 gets a 15-dimension vector from a channel of one epoch. As six channels were used, the final feature vectors were of 90-dimension. For consistency, xD also transformed each epoch into a 90-dimension feature vector. The three different algorithms produced the feature vectors of same dimension. It is to eliminate the possibility that the difference of their performances stems from the dimension distinction of feature vector.

**Figure 2:**
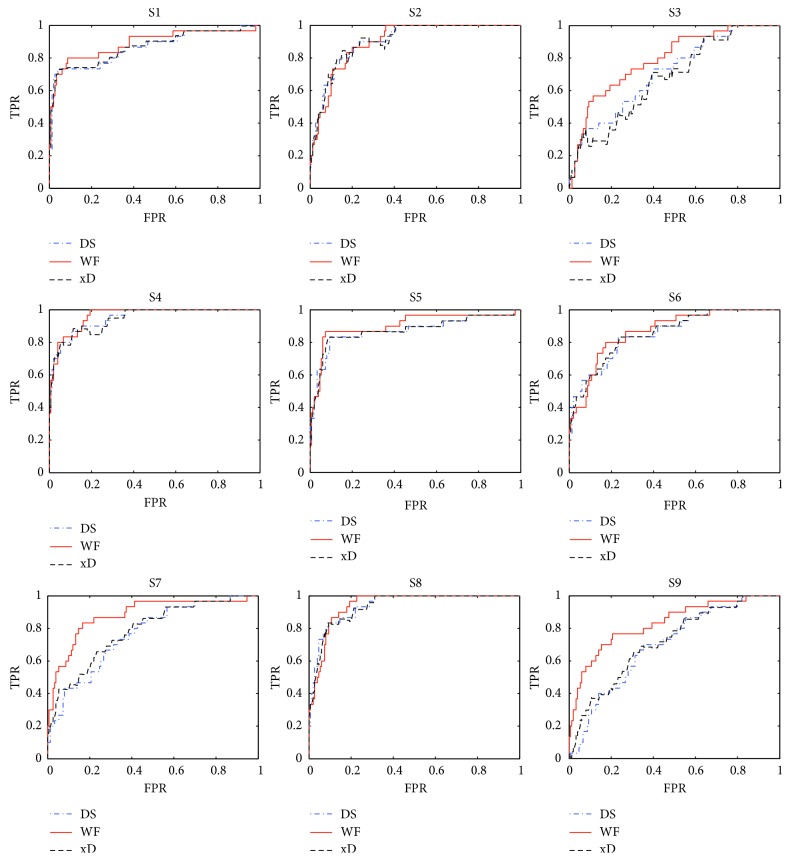
ROC curves of subject 1–9.

The experiment design has shown that a sequence, through which a subject input a character, includes 15*∗*12 EEG epochs. We investigated the classification performance by using WF, DS, and xD to classify all epochs of each sequence. For a sequence, three receiver operating characteristics (ROC) [29] curves were drawn, respectively, for WF, DS, and xD, and the areas under the ROC curve (AUC) [29] were also worked out.  Figure 2 demonstrates a ROC graph for each subject. TPR means true positive rate, and FPR means false positive rate. Every graph contains three ROC curves: WF, DS, and xD. They are the results of classification performance of WF, DS, and xD in a sequence of the subjects.

For a ROC curve, the AUC indicates the performance of classification. Bigger means better. In Figure 2, it is obvious that the WF curves have bigger AUC than those of DS and xD for S1, S3, S4, S5, S7, and S9. Their AUCs are very similar for S2, S6, and S8. Clearly, WF performs better in the example.

**Figure 3:**
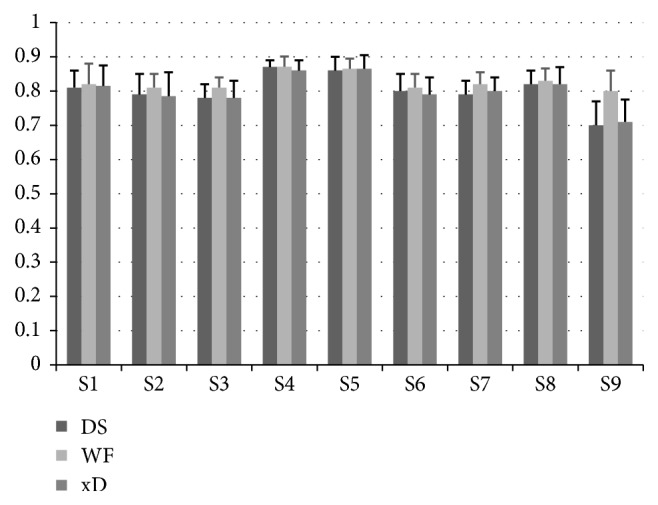
AUC means and standard deviations.

However, an example is not enough. We figured out all sequences AUCs of each subject. Their means and standard deviations are shown in Figure 3. The heights of the bars are the means of AUCs of Subject 1–9. The error bars represent the standard deviations of the AUCs. For S1, S2, S3, S6, S7, S8, and S9, WF AUC means are bigger than those of DS and xD. For S4 and S5, WF AUC means are similar to those of DS and xD. As for the standard deviations, no significant difference appears. Figure 3 shows that, in general, WF did better than DS and xD in classifying the EEG epochs of single trial from P300 Speller BCI. Furthermore, the WF, DS, and xD AUC of each sequence were, respectively, paired, and paired *t*-tests were conducted over the data sets of the paired AUCs. *P* value of the pair of WF and DS is 1.38E-62, that of WF and xD 1.43E-60, and that of DS and xD 0.15. It is very obvious that WF is superior to DS and xD in the task of classification. On the contrary, DS and xD had similar performance in the task.

### 3.3. Detection of Characters and Statistic Analysis

**Figure 4:**
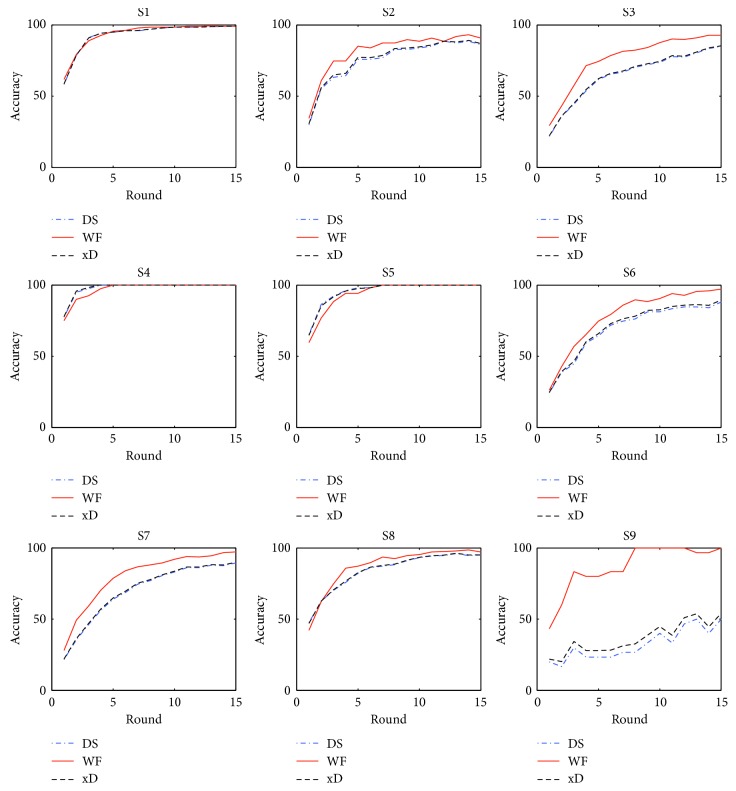
Accuracy of detecting characters.

For P300 Speller BCI, the wanted character is recognized by selecting the most possible row and column of the character matrix through the synthesis of the classification results. The accuracy of classification is not that of detecting characters. As shown in Figure 4, the accuracy of detecting characters varies basically from 20% to 75% when only the first round of EEG epochs are used. Round = *n* means that the detection of characters is the result of synthesizing the classification of the first *n*-round EEG epochs. The accuracy of detecting characters changes with the round. The curves reflect the trend that a higher accuracy could be achieved when more rounds are used. Therefore, one round of stimuli is usually not sufficient for P300 Speller BCI. By default, BCI2000 presents 15 rounds of stimuli for a wanted character. We did the experiments in the condition of BCI2000 default configuration and constructed the test data set in which every sequence contains 15 rounds of EEG epochs. Thus, we can observe how the performances of detecting characters change when the rounds vary from 1 to 15.

In Figure 4, the DS, WF, and xD curves, respectively, imply the trends that their performances change with the rounds. They can get higher accuracies when more rounds are processed. For S2, S3, S6, S7, S8, and S9, the WF curves are above the DS and xD ones, indicating that WF did better than DS and xD for these subjects. Especially, there is a big gap between the WF curves and the other two for S9, meaning that WF achieved much higher accuracies of detecting characters than DS and xD for S9. For S1, S4, and S5, the three curves are very similar, implying that they performed similarly. For the three subjects, the accuracies exceed 60% when only one round of EEG epochs is used. It is good enough. So, it is difficult for WF to obviously perform better than DS and xD for these subjects. In general, WF can clearly achieve higher accuracies of detecting characters than the other two.

Although more rounds mean higher accuracies of detecting characters, fewer rounds are expected from the perspective of efficiency. There is a trade-off between the accuracy and efficiency. To seek a good balance, we turned to the information transfer rate (ITR), a measure about the amount of communication in unit time [4]. Equation (6) and (7) show how ITR should be calculated:

(6) $$\begin{array}{c} \text{ITR}=\frac{B}{t}, \end{array}$$

(7) $$\begin{array}{c} B=\log_{2}N+P\log_{2}P+\left(1-P\right)\log_{2}\frac{1-P}{N-1}, \end{array}$$

where *N* is the number of the characters in the character matrix, *P* is the accuracy of recognizing characters, and *t* is calculated, respectively, for round = 1,2,…, 15 according to Equation (8). In Equation (8), the time unit is millisecond, and gap means the interval between the consecutive sequences. Here, we figured out *t* by setting gap as 2000 and converted the unit of *t* to minute before using *t* in the following equation:

(8) $$\begin{array}{c} t=12\times 200\times \left(\text{round}-1\right)+11\times 200+800+\text{gap}. \end{array}$$

**Figure 5:**
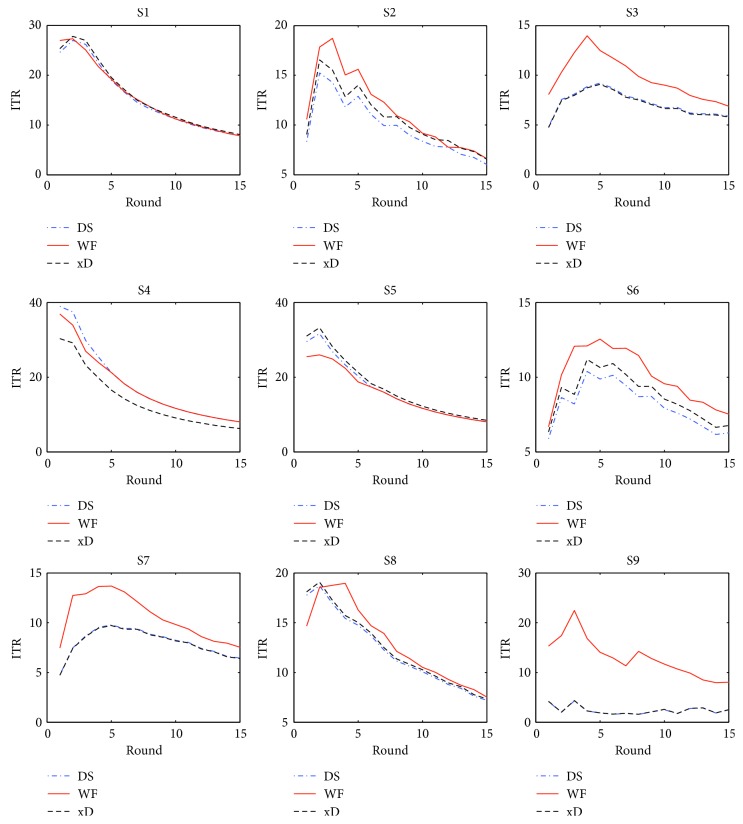
ITR curves.

The results of ITR are shown in Figure 5. The meaning of round = *n* is same as that in Figure 4. The curves reflect the trends that the ITRs change with the increase of round. Similarly, WF has higher ITRs than DS and xD for S2, S3, S6, S7, S8, and S9, and their ITRs are very high and close for S1, S4, and S5. This also means that WF is mostly superior to DS and xD. Additionally, the ITR curves can help seek the trade-off between accuracy and efficiency. We can see a peak in the ITR curves for the subjects except S4. For the WF curves, the peak is at round = 2 for S1, round = 3 for S2, round = 4 for S3, round = 2 for S5, round = 5 for S6, round = 5 for S7, round = 4 for S8, and round = 3 for S9. The peaks are the biggest ITRs for the subjects. More rounds do not lead to higher ITRs. The round numbers corresponding to the peaks are the best choices for the subjects. As for the exception of S4, the reason that no peak exists is that the accuracies of detecting characters are very high at round = 1. So, round = 1, 2, or 3 is suitable for S4 according to its accuracy curve. To sum up, WF not only significantly increases the accuracy of detecting characters but also reduces the round numbers to 2–5.

### 3.4. Speed of Parallel Computing

The parallel computing scheme is shown as Figure 1. WF was implemented in the ExtractBolts and SWLDA in the ClassifyBolts. The four kinds of computing units in the scheme were deployed on a Storm platform based on three ordinary desktop computers. The configurations of the three computers were, respectively: Intel Pentium dual core E6600 3.06 GHz, Intel Pentium CPU G850 2.90 GHz, and Intel core i5-4590 3.30 GHz. The Storm was supported by 1 G RAM on each of the three computers. The parallel computing scheme substituted for the signal processing module of BCI2000 and was connected to the other parts of BCI2000 during our online experiments. No delay cues emerged in our online experiments.

When our method is applied in P300 Speller, the system extracts the feature vector from each EEG epoch, classifies it, and begins to synthesize the classification results of all EEG epochs at the end of one round of stimuli. According to the synthesization, the system can give out the detected character or continue to present the stimuli. Whether or not the synthesization is completed in time is an important factor of the performance of our method.

**Figure 6:**
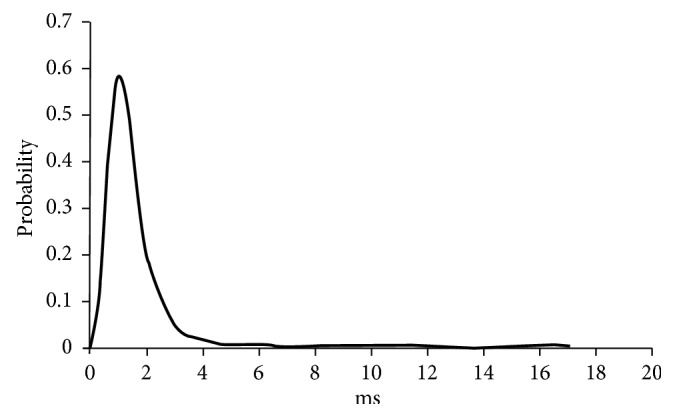
Probability distribution of the feedback time.

Under the conditions mentioned above, we tested the time from the end of one round of stimuli to the moment when the response to the round is given out. The probability distribution of the time value is shown as Figure 6. The maximum response time is 16 ms, and the mean is 1.57 ms. In P300 Speller, every stimulus lasts 200 ms. All the responses were given out before the next stimulus occurred.

## 4. Conclusions

BCI not only brings people a lot of visions about the future but also has many practical applications in the fields of rehabilitation therapy. Among all kinds of BCIs, the reliability of P300 Speller has been attracting the attention of researchers. Many efforts were made to improve P300 Speller. This work aims at developing a new feature extraction and its parallel computing scheme for P300 Speller.

The proposed feature extraction is based on Wavelet Transform, which has been proved by many researches to be a good tool for analyzing P300 component. In P300 Speller, both target EEG epochs and nontarget EEG ones contain P300 component. The difference between the two is not obvious. We mapped the EEG epochs to the wavelet space and measured the differences between target and nontarget in the space according to Fisher Criterion. The wavelet bases corresponding to small differences were filtered, and a sparse wavelet space was constructed for each subject. The feature extraction algorithm based on the sparse wavelet spaces was developed for P300 Speller.

The test results show the superiority of the proposed feature extraction. Firstly, WF, DS, and xD were applied in the classification of single-trial epochs from P300 Speller. Their performances of classifying single trial epochs were measured by AUC. The comparison of AUC between the methods indicated that WF outperformed the other two in classifying single trial epochs from P300 Speller. Secondly, WF, DS, and xD were further compared by detecting the wanted characters. For most subjects, the accuracy curves of WF are above the ones of the other two, implying that WF achieved better performance in the detection of characters of P300 Speller than DS and xD did. Finally, ITRs were calculated on basis of the detection accuracies. The comparison of ITRs demonstrated the result that is consistent with the comparison of the detection accuracies.

Additionally, the parallel computing scheme of the feature extraction was designed and implemented to ensure the fast feedback for online P300 Speller experiments. It is worth mentioning that the parallel computing scheme is able to support any other algorithms for P300 Speller. An algorithm can be implemented on our parallel computing scheme to extract the features or classify the EEG epochs for online P300 Speller experiments, even though it is computationally complex.

On the contrary, something should be further handled. The problem on which kind of wavelet bases is the best choice for our method is needed to be systematically studied. The matrix used to extract features in WF is obtained by a supervised training. Some issues on the supervised training remain unknown. For example, how much data is enough for the supervised training? When the supervised training is needed to be conducted again to adapt to the change of EEG. We plan to study such interesting problems in the future.

## Figures and Tables

**Algorithm 1:**
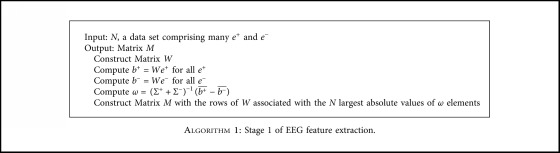
Stage 1 of EEG feature extraction.

## Data Availability

The P300 Speller BCI data used to support the findings of this study have been deposited in the Weiyun repository. Anyone can access the data by using the link https://share.weiyun.com/5Xla8up.
